# Eosinophilic gastroenteritis with negative endoscopic biopsy and no peripheral eosinophilia: A case report

**DOI:** 10.1097/MD.0000000000043289

**Published:** 2025-07-18

**Authors:** Yukai Chen, Keke Sun, Linbo Chen, Dewen Lu, Chihong Shi, Qi Lin

**Affiliations:** a Department of Gastroenterology, The Affiliated People’s Hospital of Ningbo University, Ningbo, Zhejiang Province, China.

**Keywords:** ascites, biopsy, diagnosis, eosinophilic gastroenteritis

## Abstract

**Rationale:**

Eosinophilic gastroenteritis (EoGE) is a rare inflammatory disease that can affect the entire gastrointestinal tract. Klein et al classified EoGE into 3 distinct subtypes according to the depth of eosinophilic infiltration: mucosal, muscular, and serosal.

**Patient concerns:**

We herein report a case of a 29-year-old woman who presented with abdominal pain, nausea, and vomiting, with no history of adverse reactions to any allergens. Laboratory test results revealed a normal count of peripheral blood eosinophils. Ultrasound and computed tomography revealed thickened intestinal walls and ascites in both the abdominal cavity and the pelvic cavity. Multiple endoscopic biopsies targeting several swelling segments of the intestinal wall revealed no increase in the number of eosinophils.

**Diagnoses:**

The constellation of clinical, laboratory, radiological findings, histopathological data, and the excellent response to corticosteroids led to a diagnosis of EoGE for the patient.

**Interventions:**

The patient was treated with corticosteroids.

**Outcomes:**

Symptoms improved rapidly, and the thickened intestinal walls returned to a typical level.

**Lessons:**

EoGE should be considered scrupulously, even if the count of eosinophils is in the normal range and the endoscopic biopsy is negative. Obtaining pathology of ascites as much as possible and taking a multisite biopsy sample, including both targeted and random biopsies, may improve the diagnostic rate.

## 1. Introduction

Eosinophilic gastroenteritis (EoGE) is a rare inflammatory disease, characterized by infiltration of eosinophils, which can affect the entire gastrointestinal (GI) tract.^[1]^ Kajiser first described the disease in 1937, and there were only about 300 cases reported between 1937 and 2016.^[2]^ In 1970, Klein et al classified EoGE into 3 distinct subtypes according to the depth of eosinophilic infiltration: mucosal, muscular and serosal. Because the symptoms of EoGE are similar to other GI diseases, the diagnosis of EoGE requires high sensitivity in the clinic.^[3]^ We herein describe the case of a patient who suffered from EoGE presenting as edema and exudation of the gastrointestinal wall but with no positive biopsy. The results of ascites examination guide the diagnosis. Based on our case, systematic and effective biopsy sampling in EoGE is indispensable.

## 2. Case report

A 29-year-old woman presented to our unit with complaints of recurrent abdominal pain lasting for 4 months, with nausea and vomiting for the past 3 days. The patient gave no history of previous abdominal surgery, recent travel, or adverse reactions to any allergens. She had no medical problems in previous history.

On examination we found the patient to be totally well; body temperature was 37.3°C, blood pressure 133/84 mm Hg, heart rate 92 beats/min, oxygen saturation 99%. Her abdomen was tender with no abdominal tenderness.

**Figure 1.**
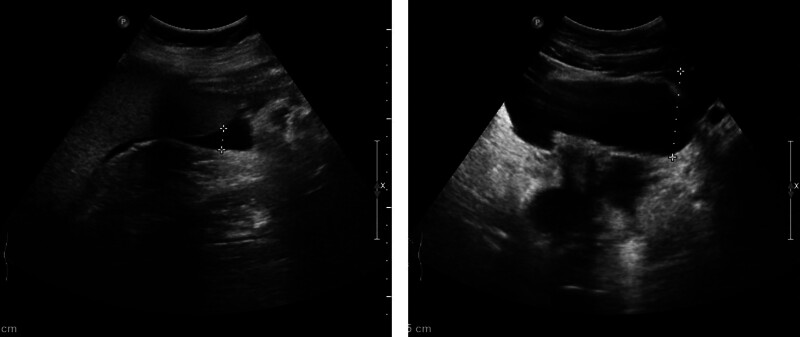
Ultrasound images. Ultrasound showed ascites in the both abdominal cavity and pelvic cavity.

**Figure 2.**
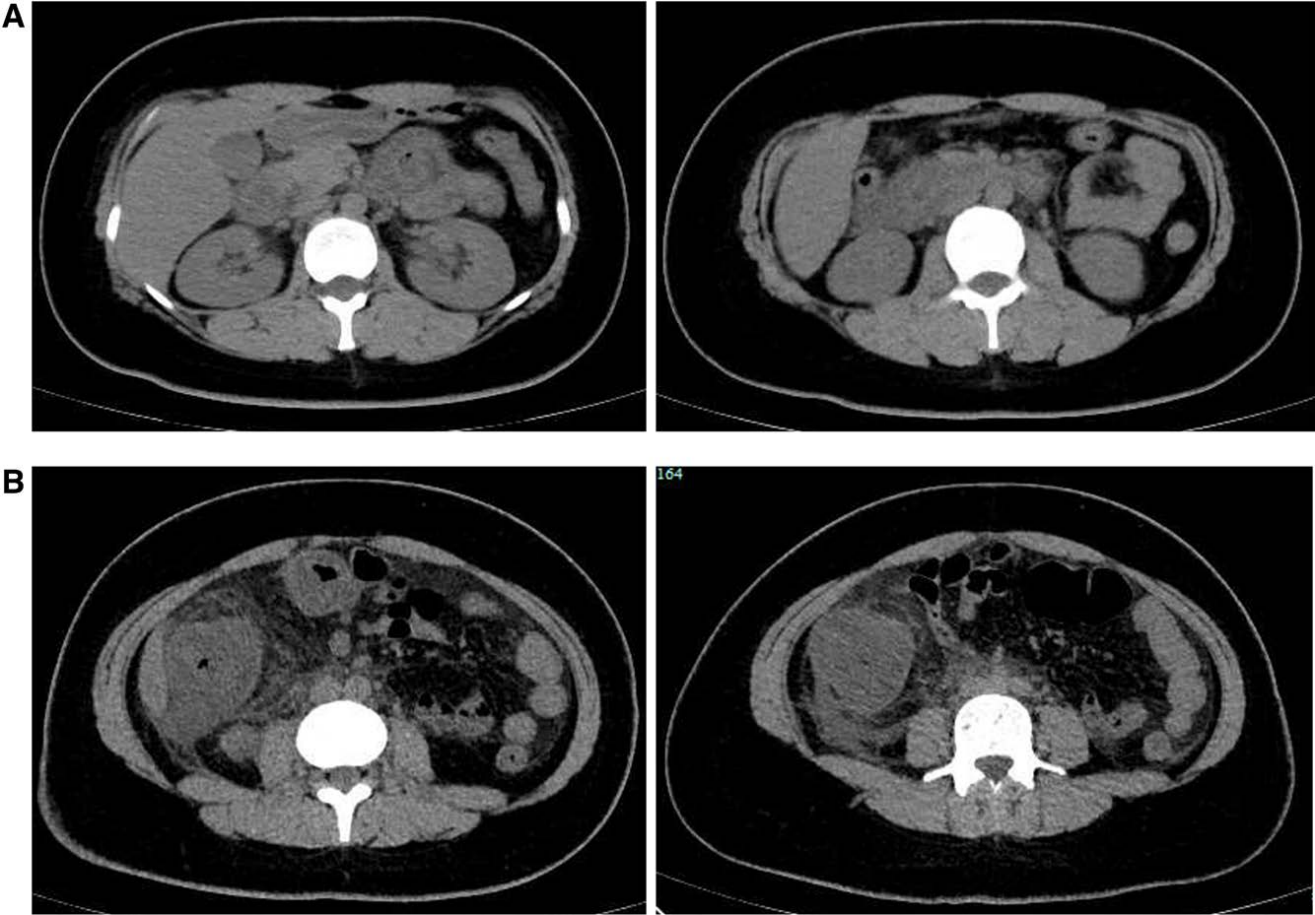
Abdominal computed tomography scan images: (A) demonstrated diffuse thickening and edema involving the gastric antrum, duodenum and partial small intestinal; (B) demonstrated thickening and edema of the ascending colon.

**Figure 3.**
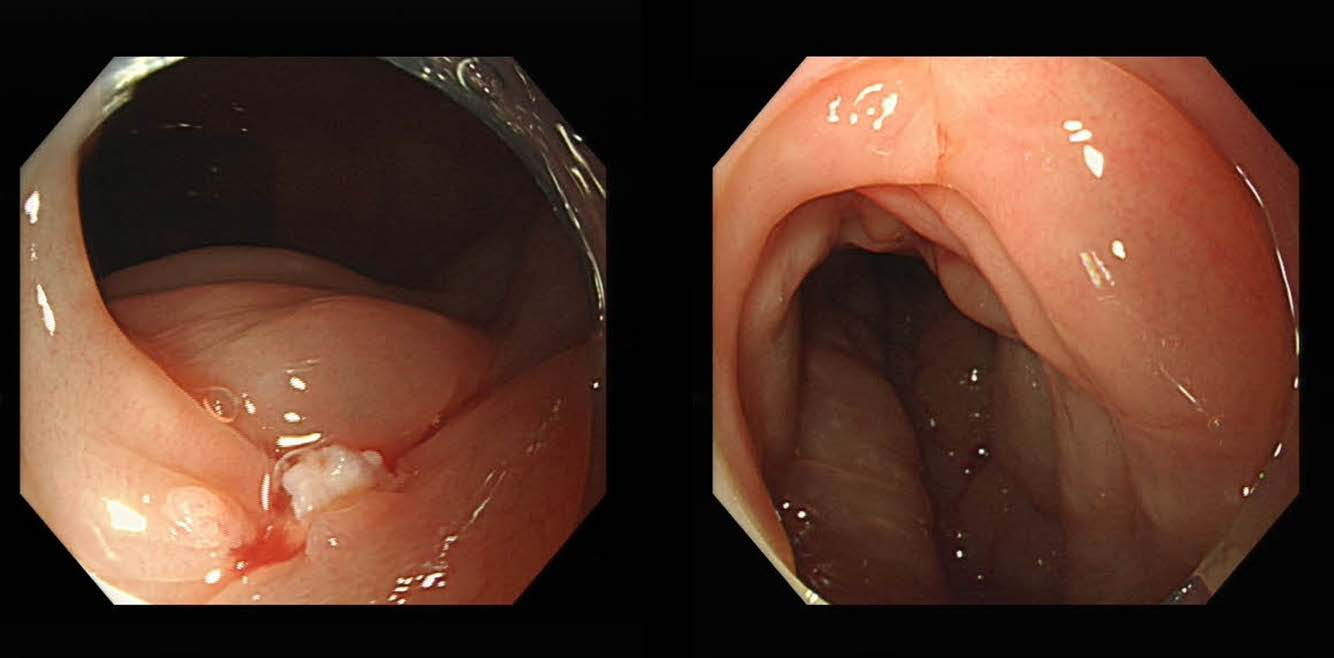
Endoscopic appearance. Colonoscopy results showed extensive swelling of the intestinal wall.

**Figure 4.**
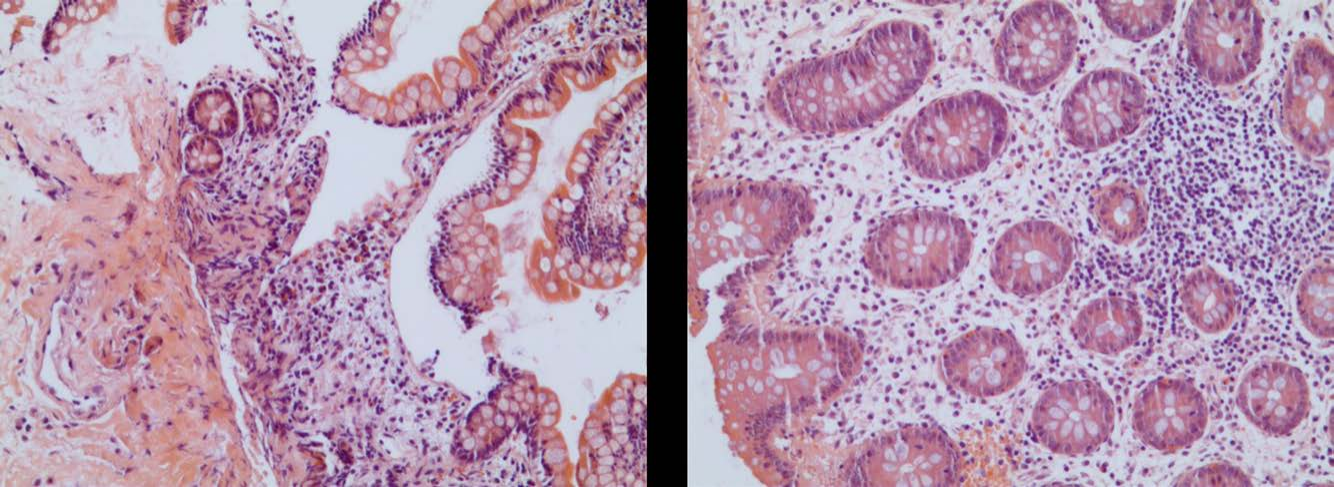
Histological examination. Biopsies results showed eosinophilic infiltration <20 eosinophiles per high-power field. Hematoxylin and eosin stain.

**Figure 5.**
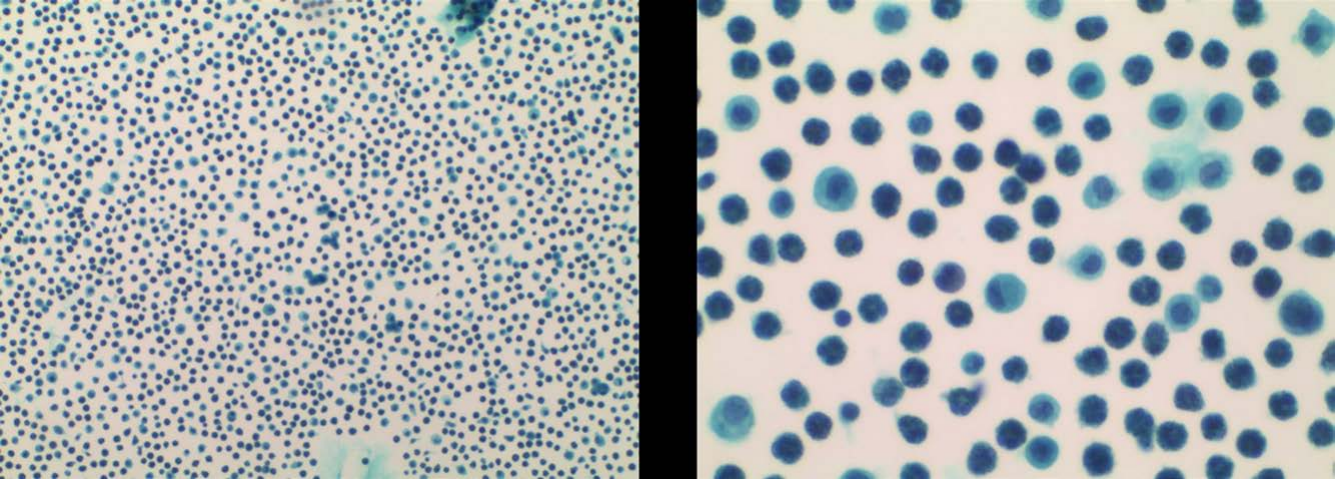
The pathology smear of ascites showed significant eosinophilic infiltration. Hematoxylin and eosin stain.

Laboratory investigations were notable for a raised IgE concentration (346 IU/mL; normal 0–100) and a slightly elevated C-reactive protein concentration (13 mg/L; normal 0–6). Leukocyte count was elevated (10.7 × 10^9^ per L; normal 3.5–9.5), while eosinophil count was 0.3 × 10^9^ per L (normal 0–0.5). Total antinuclear antibodies concentration was 1:1000, while other tests for autoimmune diseases found nothing abnormal. All tumor markers were negative. Ultrasound showed thickened intestinal walls and ascites in the both abdominal cavity and pelvic cavity (Fig. 1). An abdominal computed tomography performed on February 13 revealed diffuse thickening and edema involving the gastric antrum, duodenum and partial small intestinal (Fig. 2A). Moreover, A computed tomography performed 1 week later revealed thickening and edema of the ascending colon while the process of intestinal walls mentioned above improved (Fig. 2B). A case discussion was conducted, and the eosinophilic gastroenteritis was possibly considered to be. Thus, blood and stool analyses, including specific serological examinations and cultures, exclude viral, bacterial, and parasitic infections. The patient had a gastrointestinal endoscopy which showed extensive swelling of the intestinal wall (Fig. 3). We obtained biopsies form the obviously swollen segments, as follows: 2 biopsies from gastric antrum, 1 biopsy from duodenum, 2 from terminal ileum, 3 from ascending colon and 6 from transverse colon, including several deep biopsies. Nevertheless, large biopsies of gastroduodenal and colonic mucosal had no positive indication: normal eosinophilic infiltration with <20 eosinophiles per high-power field (Fig. 4). Fortunately, we reevaluated the pathology smear of ascites found 2 months ago, which revealed significant eosinophilic infiltration (Fig. 5). Meanwhile, we ruled out associated vasculitis.

The patient received intravenous hormonal therapy at first. Subsequently, oral maintenance therapy was initiated (Table 1). The patient’s condition stabilized and her symptoms improved rapidly; the thickened intestinal walls returned to a typical level. At the first outpatient follow-up 1 week later, the patient no long had any abdominal pain or nausea. As the patient said, she felt completely recovered and had understood such disease through this successful treatment.

**Table 1:** The dose, type and duration of corticosteroid therapy.

Date (2024)	Medicine	Dosage (mg)	Usage
February 24 to February 26	Methylprednisolone sodium succinate	40	ivgtt
February 27 to March 4	Prednisone tablets	30	po
March 5 to March 11	Prednisone tablets	25	po
March 12 to March 18	Prednisone tablets	20	po
March 19 to March 25	Prednisone tablets	15	po
March 26 to April 1	Prednisone tablets	10	po
April 2 to April 8	Prednisone tablets	5	po

## 3. Discussion

Eosinophilic gastrointestinal disorders are a spectrum of rare and heterogeneous diseases characterized by dense infiltration of eosinophils into different layers of the GI tract in the absence of secondary causes of eosinophilia.^[4,5]^ The etiology and pathogenesis of EoGE remain obscure. Research showed that bacterial infections, hygiene status and genetic and environmental may all contribute to the etiology of EoGE.^[6–8]^ Thus, a history of atopy has been reported in half of the patients with EoGE.^[9]^ Though EoGE is accepted as a Th2-mediated allergic reaction, the pathogenesis of EoGE remains complicated, as many factors can lead to eosinophil infiltration.^[10]^

Although EoGE is an uncommon disease, there are increasingly incidence and prevalence. The recent prevalence in western countries is from 5 to 8 per 1,00,000.^[11]^ EoGE can occur at any age from childhood to adulthood, in which most are diagnosed between the third and fifth decades (with a predominance in females) and is more common in the female patients.^[12]^

The clinical presentations of EoGE are nontypical and differ depending on the location and depth of infiltration of eosinophils. In all 3 Klein classifications, the mucosal subtype is predominant. Patients usually present with general abdominal pain, nausea, vomiting, weight loss diarrhea and even gastrointestinal bleeding.^[13]^ The muscular subtype results in bowel wall thickening and impaired intestinal motility, which may lead to symptoms of intestinal obstruction.^[12]^ The serosal subtype is in only 10% reported cases, presenting with eosinophilic abdominal ascites.^[14]^ In addition, this subtype has a better response to steroid therapy, which is the most widely prescribed treatment.^[12,15]^ These 3 subtypes can also exist together.

In 1990, Talley et al^[9]^ proposed the following 3 diagnostic criteria: presence of gastrointestinal symptoms; histopathological findings of eosinophilic infiltration or characteristic radiological findings with peripheral eosinophilia; and no evidence of parasitic or extraintestinal disease. To date, however, no consensus has been reached for EoGE diagnosis. There are approximately 20% patients of EoGE having no elevated level of peripheral blood.^[10,16,17]^ The most common abnormal endoscopic findings are not specific for EoGE, such as erosion, edema or erythema, and many may appear normal.^[18–20]^ Diagnosing EoGE therefore strongly relies on performing biopsies. Eosinophil infiltration can present in the stomach, intestine and colon of normal individuals, consequently, EoGE patients can exhibit the normal presence of eosinophils.^[21–23]^ Both peripheral eosinophilia and biopsy play key roles in diagnosis of EoGE. In clinical practice, insufficient biopsy sampling might produce false-negative results and missed diagnosis. Many researches have highlighted the importance of obtaining multiple biopsy samples from multiple anatomic locations, including targeted biopsies and random biopsies.^[21,24–28]^ The optimal number and location of biopsies needed to establish the diagnosis remain uncertain. Some authors suggested that at least 5 to 6 biopsies were warranted per site.^[12,29,30]^ A retrospective US study of 509 patients showed the number of biopsy fragments ranged from 4 to 7.^[31]^ What’s more, a study demonstrated that at least 8 gastric biopsies and 4 duodenal biopsies was required to diagnose.^[20]^ In addition, performing at least 2 biopsies for each of the terminal ileum, ascending colon, transverse colon, descending colon, sigmoid and rectum is advised.^[32,33]^ Even though, the positive rate of biopsy for EoGE might be low.^[31,34]^ Laparoscopic full-thickness biopsy can be helpful in establishing the diagnosis.^[23,35,36]^

We present a case of a female patient with recurrent abdominal pain accompanied by nonspecific symptoms of nausea and vomiting, with obviously thickening of the gastrointestinal tract, though, without peripheral eosinophilia and positive biopsies. In our case, we gain insufficient biopsy samples by just performing biopsies in the most severe lesion. We assume that the congestion and edema might increase the difficult in obtaining positive biopsy, and multiple biopsy samples from other random or normal segments could be helpful. In all, combining the constellation of the clinical, laboratory, radiological findings, histopathological data, and the excellent response to corticosteroids, we confirm the diagnosis.

## 4. Conclusion

EoGE is a relatively rare chronic inflammatory disease with the nonspecific symptoms, and widely adopted diagnostic criteria is necessarily needed. According to the case, EoGE should be considered scrupulously even if the count of eosinophils is in the normal range and the endoscopic biopsy is negative. There still calls for further study to have a better understanding of the EoGE.

## Author contributions

**Conceptualization:** Qi Lin.

**Investigation:** Linbo Chen, Chihong Shi.

**Project administration:** Qi Lin.

**Resources:** Qi Lin.

**Supervision:** Keke Sun, Dewen Lu.

**Writing – original draft:** Yukai Chen.

**Writing – review & editing:** Keke Sun.
